# Editorial: Aortopathy in congenital heart disease

**DOI:** 10.3389/fcvm.2023.1231646

**Published:** 2023-07-12

**Authors:** Constance G. Weismann, Joanna Hlebowicz

**Affiliations:** ^1^ Clinical Sciences Lund, Lund University, Lund, Sweden; ^2^ Department of Pediatric Cardiology, Skåne University Hospital, Lund, Sweden; ^3^ Department of Pediatric Cardiology and Pediatric Intensive Care, Ludwig Maximilian University, Munich, Germany; ^4^ Department of Cardiology, Skåne University Hospital, Lund, Sweden

**Keywords:** aortopathy, congenital heart disease, aortic stiffness, bicuspid aortic valve, coarctation of aorta, Marfan syndrome, cardiovascular risk factor

**Editorial on the Research Topic** Aortopathy in congenital heart disease

Aortopathies may be syndromic like in Marfan syndrome (MFS), non-syndromic or associated with congenital heart diseases (CHD) such as coarctation of the aorta (CoA) or bicuspid aortic valve (BAV) (1, 2). The purpose of this editorial to the research topic “Aortopathies in Congenital Heart Disease” is to summarize the data published in this chapter, and also shed more light on the clinical implications of aortopathies in CHD, particularly in relation to aortic stiffening and the potential effects of modifiable risk factors on long term outcome.

The articles included in this research topic cover a wide range of specialties within the area, ranging from molecular genetics and immunohistochemistry to clinical analyses of outcome and a case report. Briefly, the following key findings are presented in this issue:

1. Soto-Navarrete et al. demonstrated in a hamster model, that the genetic background may be a better predictor of aortopathy than the presence or absence of a BAV itself. The authors found that aortic diameter, smooth muscle apoptosis, elastic waviness, and Tgf-β and Fbn-2 expression were significantly increased in T strain animals (which have a 40% prevalence of BAV) compared to controls—irrespective of whether the aortic valve was bicuspid or tricuspid. The authors conclude that patients with the same genetic defect but differing valve morphology may carry the same risk for developing bicuspid aortopathy.
2. Robertson et al. found that patients with Loeys-Dietz syndrome and non-syndromal heritable aortopathies are often diagnosed late and have a higher risk of initial presentation with aortic dissection, when compared to MFS. At 10 years follow-up though, actuarial mean survival was not significantly different between different types of congenital aortopathies, highlighting the importance of structured aortopathy follow-up programs and preventive measures.
3. Grewal et al. described important similarities in the pathogenesis of thoracic aortic aneurysms in BAV and MFS, e.g., the intima was thinner in both BAV and MFS patients compared to controls. The authors suggest that common mechanisms should be further investigated in an effort to personalize treatment strategies.
4. Dolmaci et al. evaluated coronary arteries of a total of 90 patients with MFS, BAV and controls. They found that MFS had fewer typical cardiovascular risk factors than BAV and controls. Importantly, the prevalence of obstructive coronary sclerosis was significantly lower in MFS and BAV patients compared to controls. These findings provide a clinical correlation to above described molecular biologic findings by Grewal et al.
5. Lastly, Verheijen et al. presented an example of successful percutaneous management of an adult with an unusual presentation of CoA.

The articles in this research topic illustrate that understanding aortopathies at various levels is an important first step to personalized medicine, leading ultimately to improved outcome. Aortopathies should be viewed in the context of traditional cardiovascular risk factors and their effect on outcome in this patient population. The data so far is scarce though, which is why we are taking this opportunity to briefly review data on arterial stiffness and acquired cardiovascular risk factors in congenital aortopathies and CHD—and how these are related to outcome. We hope that this may stimulate further research in this area:

## Background on arterial stiffness and arterio-ventricular interaction

In young and healthy people, the aorta has a well-functioning Windkessel effect. Thereby, pulsatile blood flow is transformed into a steady flow supplying peripheral tissues, which enables a steady blood supply of the organs (esp. coronary arteries), reduces cardiac afterload and reduces pulsatile stress on the distal vasculature (3, 4). Arterial-ventricular interaction describes the effect of arterial stiffening on left ventricular function. With increased arterial stiffness, the pulse wave velocity increases. Thus, the reflected wave returns to the heart early (i.e., in late systole). This increases late systolic afterload, which affects thick-thin myofilament interactions and cross-bridge dissociation, leading to impaired relaxation, an important part of diastole (5). As a result, systolic or diastolic heart failure may evolve.

## Arterial stiffness in congenital heart disease

Arterial stiffness is a well-known predictor of cardiovascular morbidity and mortality in the general population (6). It has been extensively studied in adults with traditional cardiovascular risk factors such as, e.g., diabetes, dyslipidemia or hypertension, which are typically acquired as mid-aged adults. Though there is evidence that several types of CHD or genetic connective tissue disorders are associated with increased arterial stiffness, the etiology, extent, physiologic impact, changes with age and prognostic implications of vascular changes in this context are not well understood to date (7).

The most well studied congenital lesions in the field of arterial stiffness are BAV, CoA, and MFS—all of which are associated with intrinsic aortic wall abnormalities that extend beyond the actual anatomic lesion of aortic dilation vs. narrowing (8, 9). Over the last two decades, it has become evident that these histologic abnormalities translate to increased arterial stiffness on a physiologic level (10–12).

Aortic stiffening (and often dilation) in these groups starts during childhood and progresses with age. While MFS patients have general aortic stiffening together with thinning of the common carotid intima-media, this is not seen in BAV patients who have proximally increased aortic stiffness and pulse wave reflection (13–16). Similarly, on a histologic level, MFS and BAV patients have thinner intima, a lower expression of contractile vascular smooth muscle cells, and more elastic fiber thinning compared to controls (Grewal et al.). However, other features of cardiovascular ageing differed between the BAV and MFS in this study (Grewal et al.). Interestingly, there is also evidence that the risk for obstructive coronary atherosclerosis is decreased rather than increased in both MFS and BAV patients alike, supporting the above functional, structural and histologic findings (Dolmaci et al.).

Linking outcome to micro- and macro scalar arterial characteristics in CHD patients with aortopathies would be an important step to personalized medicine. Ultimately, longitudinal follow-up will be crucial in determining changes in arterial stiffness, central blood pressure and diastolic function over time and in response to changes in medical management.

## Acquired cardiovascular risk factors and outcome in CHD

Survival of patients with MFS has increased throughout the last few decades (17, 18). Likewise, improved treatment options for patients with CHD have led to an increasing number of children surviving well into adulthood, i.e., they are exposed to the adverse effects of increased arterial stiffness much longer than the average adult with atherosclerotic cardiovascular disease (19). At the same time, adults with CHD are more likely to have typical acquired cardiovascular risk factors (20). Most importantly, they have a strikingly increased risk for cardiovascular events, i.e., adults with CHD with ≤2 cardiovascular risk factors have more than twice the risk for major adverse cardiovascular events compared to non-CHD adults with ≥5 risk factors (20).

Among CoA patients, e.g., the risk for acquired cardiovascular risk factors appears particularly high (21, 22). Moreover, in MFS the body mass index may not be a good predictor of overweight and obesity as the relative muscle mass is decreased and the proportion of fat is increased (23). Additionally, overweight or obese MFS patients may be at increased risk for adverse aortic events (24).

To date, many aortopathy patients are still restriced from exercise, even though the European Society of Cardiology (ESC) has published exercise recommendations specific for the type of underlying aortopathy and degree of aortic dilation (25). In fact, limited mouse and human data suggests a beneficial effect of aerobic training on aortic root growth without major adverse events (26–28). If aortopathy patients were more physically active, this would likely increase muscle mass and lower the risk of overweight and obesity and perhaps, preventing the development of secondary cardiovascular risk factors.

Thus, over the last 50 years, the focus has shifted from improving early survival to optimizing long term outcome. Minimizing acquired cardiovascular risk factors plays an increasing role in the counseling of patients with CHD associated aortopathies.

## Conclusion

Aortopathies in CHD and genetic aortopathies are heterogeneous with variable mechanisms for increased arterial stiffness and varying risks for aortic complications. What all aortopathy patients have in common though is that they are exposed to increased arterial stiffness for a life-time—as opposed to the typical patient with acquired cardiovascular disease. The combination of a congenital aortopathy and acquired cardiovascular risk factors may be a particularly risky combination. Further research is needed to identify personalized treatment strategies for patients with aortopathies, taking into account their individual risk factors. Perhaps most importantly, we as medical professionals need to focus more on minimizing additional cardiovascular risk factors from an early age by encouraging a healthy lifestyle and exercise.

## References

[1] Milewicz DM Braverman AC De Backer J Morris SA Boileau C Maumenee IH Marfan syndrome. Nat Rev Dis Primers. (2021) 7:64. 10.1038/s41572-021-00298-7 34475413 PMC9261969

[2] Cotts TB Salciccioli KB Swanson SK Yetman AT. Aortopathy in congenital heart disease. Cardiol Clin. (2020) 38:325–36. 10.1016/j.ccl.2020.04.002 32622488

[3] Safar ME Levy BI Struijker-Boudier H. Current perspectives on arterial stiffness and pulse pressure in hypertension and cardiovascular diseases. Circulation. (2003) 107:2864–9. 10.1161/01.CIR.0000069826.36125.B4 12796414

[4] Tomiyama H Yamashina A. Non-invasive vascular function tests: their pathophysiological background and clinical application. Circ J. (2010) 74:24–33. 10.1253/circj.CJ-09-0534 19920359

[5] Borlaug BA Melenovsky V Redfield MM Kessler K Chang HJ Abraham TP Impact of arterial load and loading sequence on left ventricular tissue velocities in humans. J Am Coll Cardiol. (2007) 50:1570–7. 10.1016/j.jacc.2007.07.032 17936156

[6] Vlachopoulos C Aznaouridis K Stefanadis C. Prediction of cardiovascular events and all-cause mortality with arterial stiffness: a systematic review and meta-analysis. J Am Coll Cardiol. (2010) 55:1318–27. 10.1016/j.jacc.2009.10.061 20338492

[7] Sandhu K Pepe S Smolich JJ Cheung MMH Mynard JP. Arterial stiffness in congenital heart disease. Heart Lung Circ. (2021) 30:1602–12. 10.1016/j.hlc.2021.07.018 34420886

[8] Isner JM Donaldson RF Fulton D Bhan I Payne DD Cleveland RJ. Cystic medial necrosis in coarctation of the aorta: a potential factor contributing to adverse consequences observed after percutaneous balloon angioplasty of coarctation sites. Circulation. (1987) 75:689–95. 10.1161/01.CIR.75.4.689 2951035

[9] Nataatmadja M West M West J Summers K Walker P Nagata M Abnormal extracellular matrix protein transport associated with increased apoptosis of vascular smooth muscle cells in Marfan syndrome and bicuspid aortic valve thoracic aortic aneurysm. Circulation. (2003) 108(Suppl 1):II329–34. 10.1161/01.cir.0000087660.82721.15 12970255

[10] Hirata K Triposkiadis F Sparks E Bowen J Wooley CF Boudoulas H. The Marfan syndrome: abnormal aortic elastic properties. J Am Coll Cardiol. (1991) 18:57–63. 10.1016/S0735-1097(10)80218-9 2050942

[11] de Divitiis M Pilla C Kattenhorn M Zadinello M Donald A Leeson P Vascular dysfunction after repair of coarctation of the aorta: impact of early surgery. Circulation. (2001) 104:I165–70. 10.1161/hc37t1.094900 11568050

[12] Tzemos N Lyseggen E Silversides C Jamorski M Tong JH Harvey P Endothelial function, carotid-femoral stiffness, and plasma matrix metalloproteinase-2 in men with bicuspid aortic valve and dilated aorta. J Am Coll Cardiol. (2010) 55:660–8. 10.1016/j.jacc.2009.08.080 20170792

[13] Weismann CG Hlebowicz J Akesson A Liuba P Hanseus K. Comprehensive characterization of arterial and cardiac function in Marfan syndrome-can biomarkers help improve outcome? Front Physiol. (2022) 13:873373. 10.3389/fphys.2022.873373 35547588 PMC9081671

[14] Voges I Kees J Jerosch-Herold M Gottschalk H Trentmann J Hart C Aortic stiffening and its impact on left atrial volumes and function in patients after successful coarctation repair: a multiparametric cardiovascular magnetic resonance study. J Cardiovasc Magn Reson. (2016) 18:56. 10.1186/s12968-016-0278-6 27618813 PMC5020476

[15] Weismann CG Maretic A Grell BS Akesson A Hlebowicz J Liuba P. Multimodal assessment of vascular and ventricular function in children and adults with repaired aortic coarctation. Int J Cardiol. (2021) 323:47–53. 10.1016/j.ijcard.2020.08.095 32889020

[16] Weismann CG Ljungberg S Akesson A Hlebowicz J. Multimodal assessment of vascular and ventricular function in children and adults with bicuspid aortic valve disease. Front Cardiovasc Med. (2021) 8:643900. 10.3389/fcvm.2021.643900 33834044 PMC8021774

[17] Silverman DI Burton KJ Gray J Bosner MS Kouchoukos NT Roman MJ Life expectancy in the Marfan syndrome. Am J Cardiol. (1995) 75:157–60. 10.1016/S0002-9149(00)80066-1 7810492

[18] Vanem TT Geiran OR Krohg-Sorensen K Roe C Paus B Rand-Hendriksen S. Survival, causes of death, and cardiovascular events in patients with Marfan syndrome. Mol Genet Genomic Med. (2018) 6:1114–23. 10.1002/mgg3.489 30393980 PMC6305663

[19] Glinianaia SV Morris JK Best KE Santoro M Coi A Armaroli A Long-term survival of children born with congenital anomalies: a systematic review and meta-analysis of population-based studies. PLoS Med. (2020) 17:e1003356. 10.1371/journal.pmed.1003356 32986711 PMC7521740

[20] Saha P Potiny P Rigdon J Morello M Tcheandjieu C Romfh A Substantial cardiovascular morbidity in adults with lower-complexity congenital heart disease. Circulation. (2019) 139:1889–99. 10.1161/CIRCULATIONAHA.118.037064 30813762 PMC7309588

[21] Fedchenko M Mandalenakis Z Dellborg H Hultsberg-Olsson G Bjork A Eriksson P Cardiovascular risk factors in adults with coarctation of the aorta. Congenit Heart Dis. (2019) 14:549–58. 10.1111/chd.12785 31099471

[22] Smith-Parrish M Yu S Rocchini A. Obesity and elevated blood pressure following repair of coarctation of the aorta. J Pediatr. (2014) 164:1074–1078.e1. 10.1016/j.jpeds.2014.01.043 24607241

[23] Suleiman MN Freilinger S Meierhofer C May M Bischoff G Ewert P The relation of aortic dimensions and obesity in adults with Marfan or Loeys-Dietz syndrome. Cardiovasc Diagn Ther. (2022) 12:787–802. 10.21037/cdt-22-383 36605074 PMC9808108

[24] Yetman AT McCrindle BW. The prevalence and clinical impact of obesity in adults with Marfan syndrome. Can J Cardiol. (2010) 26:137–9. 10.1016/S0828-282X(10)70370-6 20386774 PMC2886547

[25] Budts W Pieles GE Roos-Hesselink JW Sanz de la Garza M D'Ascenzi F Giannakoulas G Recommendations for participation in competitive sport in adolescent and adult athletes with congenital heart disease (CHD): position statement of the sports cardiology & exercise section of the European association of preventive cardiology (EAPC), the European society of cardiology (ESC) working group on adult congenital heart disease and the sports cardiology, physical activity and prevention working group of the association for European paediatric and congenital cardiology (AEPC). Eur Heart J. (2020) 41:4191–9. 10.1093/eurheartj/ehaa501 32845299

[26] Benninghoven D Hamann D von Kodolitsch Y Rybczynski M Lechinger J Schroeder F Inpatient rehabilitation for adult patients with Marfan syndrome: an observational pilot study. Orphanet J Rare Dis. (2017) 12:127. 10.1186/s13023-017-0679-0 28701211 PMC5508759

[27] Selamet Tierney ES Chung S Stauffer KJ Brabender J Collins RT 2nd Folk R Can 10 000 healthy steps a day slow aortic root dilation in pediatric patients with Marfan syndrome? J Am Heart Assoc. (2022) 11:e027598. 10.1161/JAHA.122.027598 36453629 PMC9851465

[28] Mas-Stachurska A Siegert AM Batlle M Gorbenko Del Blanco D Meirelles T Rubies C Cardiovascular benefits of moderate exercise training in Marfan syndrome: insights from an animal model. J Am Heart Assoc. (2017) 6:e006438. 10.1161/JAHA.117.006438 28947563 PMC5634291

